# Phase II trials of fosquidone, (GR63178A), in colorectal, renal and non-small cell lung cancer. CRC Phase II Clinical Trials Committee.

**DOI:** 10.1038/bjc.1992.127

**Published:** 1992-04

**Authors:** S. B. Kaye, M. Brampton, P. Harper, J. Smyth, D. J. Kerr, M. Gore, J. A. Green, E. Gilby, S. M. Crawford, G. J. Rustin

**Affiliations:** Beatson Oncology Centre, Western Infirmary, Glasgow, UK.

## Abstract

A total of 61 eligible patients with metastatic cancer have been treated in a series of Phase II trials of the novel pentacyclic pyrroloquinone, fosquidone. Tumour types were colorectal (23), renal (21), and non small cell lung (17). No patient had received prior chemotherapy. The drug was given intravenously as a 20 min infusion at the dose of 120 mg-2 on days 1 to 5 every 3 weeks. Treatment was well tolerated; the only significant side effects being mild nausea and generalised musculo-skeletal pains. Response was assessed after two cycles of therapy. No patient achieved an objective partial response. A total of nine patients demonstrated stable disease for a median duration of 11 weeks. Using this schedule of administration, fosquidone has no significant antitumour activity in this group of tumours.


Br. J. Cancer (1992), 65, 624–625 © Macmillan Press Ltd., 1992

### Phase II trials of fosquidone, (GR63178A), in colorectal, renal and non-small cell lung cancer

S.B. Kaye[1], M. Brampton[2], P. Harper[3], J. Smyth[4], D.J. Kerr[1], M. Gore[5], J.A. Green[6], E. Gilby[7], S.M. Crawford[8] & G.J. Rustin[9] on behalf of the CRC Phase II Clinical Trials Committee

[1]*Beatson Oncology Centre, Western Infirmary, Glasgow;* [2]*CRC Data Centre, Charing Cross Hospital, London;* [3]*Guy's Hospital, London;* [4]*Western General Hospital, Edinburgh;* [5]*Royal Marsden Hospital, London;* [6]*Clatterbridge Hospital, Wirral;* [7]*Royal United Hospital, Bath;* [8]*Airedale General Hospital, West Yorkshire;* [9]*Mount Vernon Hospital, London, UK.*

**Summary** A total of 61 eligible patients with metastatic cancer have been treated in a series of Phase II trials of the novel pentacyclic pyrroloquinone, fosquidone. Tumour types were colorectal (23), renal (21), and non small cell lung (17). No patient had received prior chemotherapy.

The drug was given intravenously as a 20 min infusion at the dose of 120 mg$^{-2}$ on days 1 to 5 every 3 weeks. Treatment was well tolerated; the only significant side effects being mild nausea and generalised musculo-skeletal pains.

Response was assessed after two cycles of therapy. No patient achieved an objective partial response. A total of nine patients demonstrated stable disease for a median duration of 11 weeks.

Using this schedule of administration, fosquidone has no significant antitumour activity in this group of tumours.

Fosquidone is a water soluble analogue of the drug mitoquidone, which was the first of a series of pentacyclic pyrroloquinones developed as anti-tumour agents. Mitoquidone was of particular interest because of its broad spectrum of activity in rodent solid tumours and xenografts with no demonstrable activity in P388 or L1210 leukaemia (Fenton *et al.*, 1989). The basis of its antitumour efficacy was unknown but these characteristics suggested a different mechanism of action from that of conventional cytotoxic agents. Phase I trials with Mitoquidone were closed prematurely because of solubility problems, and the possible occurrence of intravascular drug precipitation (Speth *et al.*, 1988). However antitumour efficacy had been recorded in one patient before the study closed.

Fosquidone has similar biological characteristics to the parent compound, and is much more soluble by virtue of its phosphate side chain. A particular feature of preclinical studies was its schedule-dependency in solid tumours; daily repeated i.v. administration was more effective than high dose intermittent administration. Antitumour activity in human tumour xenografts was seen in breast, ovary, colon and head and neck tumours (Fenton *et al.*, 1989).

Phase I trials with Fosquidone were conducted with three schedules; single dose weekly, daily for 5 days every 3 weeks, and three times per week for 3 consecutive weeks (Cassidy *et al.*, 1989; Smyth *et al.*, 1990; Planting *et al.*, 1990). The maximum tolerated dose was 250 mg m$^{-2}$ for the 5 day schedule; at this level headache as well as pain sometimes at the site of tumour or metastases, were dose-limiting. Minor clinical responses were reported in three patients (with lung, head and neck and colonic tumours).

The schedule which was chosen for this Phase II study was the daily for 5 days schedule, and this was selected for the following reasons.

(a) This schedule permits regular, frequent administration of Fosquidone.
(b) At this dose level in the Phase I trial toxicity was minor, comprising WHO grade 1 or 2 nausea and vomiting and grade 1 or 2 headache.
(c) At this dose level, the measured AUC after each daily dose (9–29 $\mu$g ml$^{-1}$ min$^{-1}$) was in excess of that recorded at the tumoricidal dose in the rat hepatoma model (Smyth *et al.*, 1990).

Correspondence: S.B. Kaye.
Received 4 November 1991; accepted 5 December 1991.

A Phase II programme was therefore initiated in which the Early Clinical Trials Group of the EORTC co-operated with the Phase II Trials Committee of the Cancer Research Campaign in the UK. This study reports the Phase II trials in the following tumour types performed by the CRC Phase II Committee: colon, non small cell lung and kidney.

### Patients and methods

Eligibility criteria included histologically proven and measurable and/or evaluable advanced disease, WHO performance status of 0, 1 or 2; age up to 75 years; and adequate bone marrow, hepatic and renal function. Patients should not have received prior chemotherapy.

Treatment was given as an i.v. infusion over 20 min, on days 1–5 of a 3 week cycle, at the dose of 120 mg m$^{-2}$ per day. The drug was diluted in 5% dextrose to a final concentration not exceeding 2.0 mg ml$^{-1}$.

Patients were scheduled to receive at least two courses of chemotherapy, and to assess activity for each tumour type at least 14 cases were required.

Response assessment was made by repeated clinical and radiological examinations as appropriate. Complete and partial responses, stable and progressive disease were defined by agreed WHO criteria. Patients who demonstrated evident tumour progression after one course of treatment discontinued at that stage and were classified as 'early progression'.

### Results

A total of 70 patients were entered into the Phase II trials; 26 patients with colon cancer, 18 with non small cell lung, and 26 with renal cancer. Of the 70 patients entered, nine were deemed ineligible. The reasons were inadequate bone marrow or renal function in seven, prior chemotherapy in one and no histological confirmation of cancer in one case.

The distribution of patients and details of characteristics according to tumour type are given in Table I. A total of 143 complete courses of treatment were given, and the breakdown in tumour type is also given in Table I. Of the 61 eligible patients, 22 received one course only, 16 received two courses and 23 received three or more complete courses. The maximum number received was six courses.

Responses assessed after two courses are given in Table II. Also included are 15 patients with 'early progression' after one cycle of treatment only. A further five patients were

**Table I** Patient characteristics

| | Colon | Study Non small cell lung | Renal |
|---|---|---|---|
| Number of patients | 26 | 18 | 26 |
| Number eligible | 23 | 17 | 21 |
| Sex: Male/Female | 12/11 | 14/3 | 16/5 |
| Median age (range) | 53 (40–74) | 57 (45–74) | 57 (29–72) |
| WHO Performance status | | | |
| 0 | 12 | 4 | 3 |
| 1 | 10 | 12 | 14 |
| 2 | 1 | 1 | 4 |
| Total number of completed courses | 55 | 32 | 56 |

**Table II** Response evaluation

| *Study* *Response* | *Colon* | *Non small cell lung* | *Renal* |
|---|---|---|---|
| CR | 0 | 0 | 0 |
| PR | 0 | 0 | 0 |
| NC | 5 | 1 | 3 |
| Progression | 12 | 6 | 14 |
| 'Early progression' | 4 | 7 | 4 |
| Not evaluable | 2 | 3 | 0 |

defined as 'non evaluable' because they either did not receive one complete cycle or were not adequately reassessed at the appropriate time. A total of nine patients achieved stable disease for a median duration of 11 weeks.

Treatment was well tolerated. Haematological toxicity was absent. Nausea and vomiting occurred with approximately 40% of courses, but was generally Grade 1 or 2 in severity. Musculo skeletal pain was noted with approximately 30% of courses, and 25% of courses were associated with mild or moderate headaches. In two cases treatment was stoppped because of generalised pain and headache and in one case because of chest pain, but symptoms of this severity were exceptional. Other occasional findings included irritation at the site of injection, a degree of malaise or fatigue, and exacerbation of pain at disease sites.

### Discussion

Analysis of these Phase II studies has revealed no significant activity for Fosquidone, when using a 5 daily schedule every 3 weeks, despite the fact that none of the patients treated had received prior chemotherapy.

The experimental antitumour efficacy of Fosquidone remains unexplained. The drug was inactive when tested *in vitro* in a number of cell lines, while clearly effective *in vivo* in several tumour models (Fenton *et al.*, 1989). Possible explanations could include metabolic conversion to an active species, or else the involvement of immunomodulatory or vascular mechanisms, but experimental data in support of any of these hypotheses are not yet available. Another striking feature of the drug's experimental activity is its clear schedule dependency, with long term exposure being the most effective in murine tumour models. For this reason additional Phase I trials have been initiated in an attempt to simulate the experimentally active schedules, using long term infusions and also orally formulated Fosquidone. However, further development of antitumour agents based on the pentacyclic pyrroloquinone structure may depend on a clearer understanding of the basis for its activity.

### References

CASSIDY, J., LEWIS, C., SETANOIANS, A. & 6 others (1989). Phase I trial of GR63178A. *NCI-EORTC Symposium on New Drugs Proceedings*, Abstract 91.

FENTON, R.J., KUMAR, K.A., O'SULLIVAN, S.M., NEATE, M.S., SPILLING, C.R. & KNOX, P. (1989). *In vivo* anti-tumour activity of the mitoquidone analogue, GR63178A. *NCI-EORTC New Drug Symposium Proceedings*, 1989. Abstract 89.

PLANTING, A.S., WOOTTON, C.M., STOTER, G., SMITH, R.A. & VERWEIJ, J. (1990). Phase I study of GR63178A administered three times per week. *Proc. Amer. Soc. Clin. Oncol.*, **9**, 88.

SMYTH, J.F., ECCLES, D., CUMMINGS, J., STEWART, M., CORNBLEET, M. & LEONARD, R. (1990). Phase I study of the pyrroloquinolone GR63178A. *Proc. Amer. Soc. Clin. Oncol.*, **9**, 71.

SPETH, P.A.J., GORE, M.E., PATEMAN, A.J. & 13 others (1988). Phase I and pharmacokinetic studies with the pentacyclic pyrroloquinone mitoquidone. *Cancer Chemother. Pharmacol.*, **21**, 343.


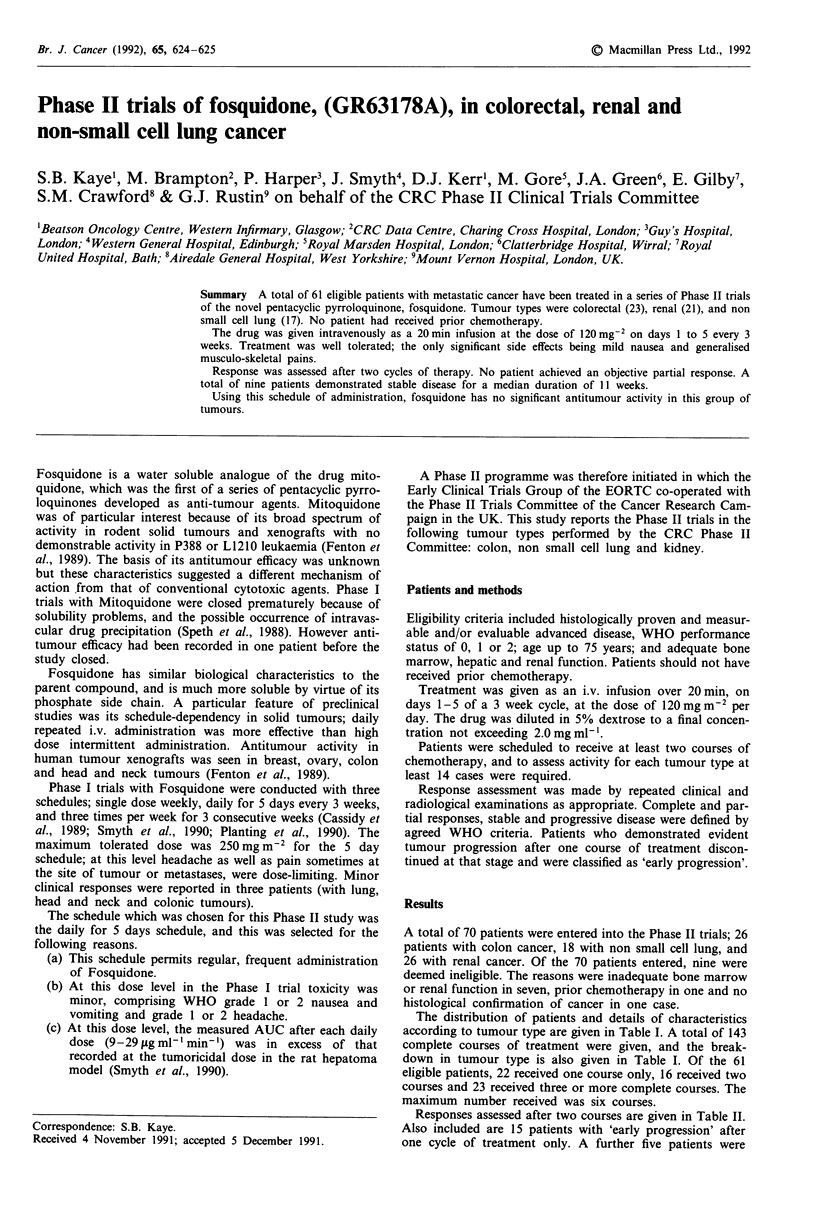

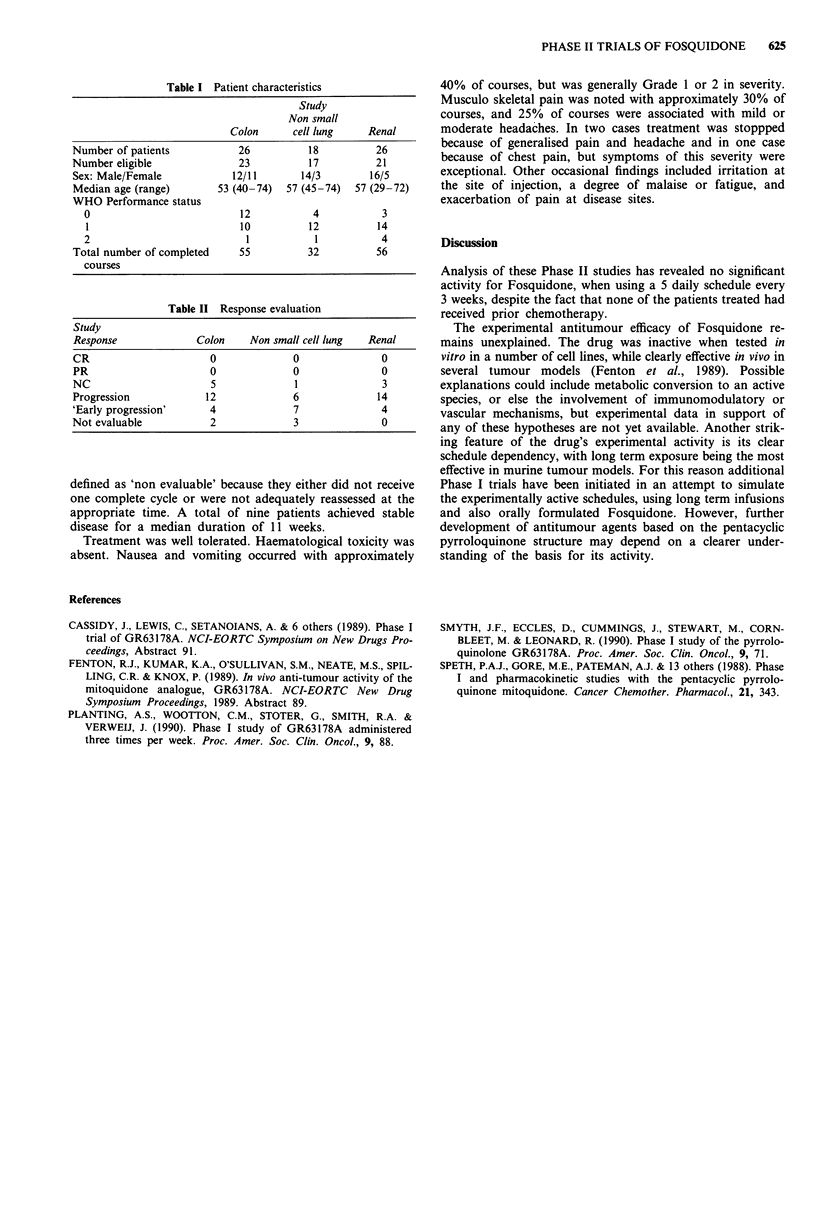


## References

[OCR_00246] Speth P. A., Gore M. E., Pateman A. J., Newell D. R., Bishop J. A., Ellis W. J., Green J. A., Gumbrell L. A., Linssen P. C., Miller A. (1988). Phase I and pharmacokinetic studies with the pentacyclic pyrroloquinone mitoquidone.. Cancer Chemother Pharmacol.

